# Green and environmentally friendly synthesis of silver nanoparticles with antibacterial properties from some medicinal plants

**DOI:** 10.1186/s12896-023-00828-z

**Published:** 2024-01-23

**Authors:** Samira Asefian, Mansureh Ghavam

**Affiliations:** Department of Nature Engineering, Faculty of Natural Resources and Earth Sciences, University of Kashan, Kashan, Iran

**Keywords:** Green synthesis, Nanotechnology, Extract, Anti-microbial activity, Wound healing

## Abstract

Recently there have been a variety of methods to synthesize silver nanoparticles, among which the biosynthesis method is more noticeable due to features like being eco-friendly, simple, and cost-efficient. The present study aims for the green synthesis of silver nanoparticles from the extract of the three plants *A*. *wilhelmsi, M*. *chamomilla,* and *C. longa*; moreover, it pledges to measure the antibacterial activity against some variants causing a skin rash. The morphology and size of the synthesized silver nanoparticles were evaluated by UV.vis, XRD, SEM, and FTIR analyses. Then results showed a color alteration from light yellow to dark brown and the formation of silver nanoparticles. The absorption peak with the wavelength of approximately 450 nm resulting from the Spectrophotometry analysis confirmed the synthesis of silver nanoparticles. The presence of strong and wide peaks in FTIR indicated the presence of OH groups. The SEM results showed that most synthesized nanoparticles had a spherical angular structure and their size was about 10 to 20 nm. The highest inhibition power was demonstrated by silver nanoparticles synthesized from the extract combined from all three species against Gram-positive bacteria *Staphylococcus aureus* and *Staphylococcus epidermidis* (23 mm) which had a performance far more powerful than the extract. Thus, it can be understood that the nanoparticles synthesized from these three species can act as potential environment-friendly alternatives to inhibit some variations causing skin disorders; an issue that calls for further clinical studies.

## Introduction

Nowadays, nanotechnology is a novel and appealing scientific and research field [1]. This technology is research and development at atomic and molecular levels resulting in the synthesis of particles with dimensions of 1 to 100 [2]. Among these, nanoparticles have vast applications in medicine, agriculture, and imaging [3, 4], due to possessing mechanical, chemical, and physical characteristics including the surface area to volume ratio [5], uniform distribution of size, electric conductivity, smaller size, higher temperature, photocatalytic activity, and biocompatibility [3, 4].

Among metal nanoparticles, silver nanoparticles have been extremely popular because of their broad applications such as antiviral, anticancer, antibacterial, catalytic, optoelectronic, and medical uses [6]. Silver nanoparticles have been also studied and synthesized in recent years to control infections and deteriorations and as a strong antibacterial substance in medicine [7, 8]. Nowadays scientists are interested in studying silver nanoparticles due to their various applications in pharmacy, cosmetics, dye degradation, calculations, and sensors [9–11].

Skin is the human’s biggest organ, covering the whole body and aiding against alien factors. When it loses its uniformity it can no longer carry out its protective activities for a person’s health. Skin damage is the main problem of the health care system worldwide because if any damage occurs to this layer, the skin loses its protective function [12]. A wound can be described as a deficiency or breakage in the skin which can be caused by physical, chemical, or thermal injury or be the result of a physiologic ailment or a pre-existing medical condition that disrupts the anatomic performance of the natural injury to the skin [13]. The colonization of microbes in the wound is one of the reasons for a delay or failure in its repair; thus, controlling the growth of organisms inside a wound will aid its repair [14]. Nanomedical equipment, especially nanoparticles bring about a hopeful future for the development of anti-microbial agents [15]. Among nanoparticles, nanosilver has become popular as an antimicrobial agent working against a wide spectrum of microbes and fungi, as well as having advanced nanoparticle qualities [16].

Plants produce secondary metabolites to protect themselves against environmental stress [17]. The use of natural products against global public health problems is prominent. Among the potentials of these products, we can mention anti-inflammatory, antioxidant, anti-depressant, anti-cancer and anti-microbial properties [18, 19]. There is a growing recognition of medicinal plants as providing practical resources for new drug molecules, especially when most common synthetic drugs fail [20].

*Achillea wilhelmsii* K.Koch is a plant of the Asteraceae family. This plant is perennial and has herbaceous growth, with a height between 15-30 cm. The leaves of this plant are white without petioles and the flowers are yellowish-white, emerging often in May and June. It has a wide distribution in various regions of Iran, especially in central and western areas [21]. This valuable medicinal plant has been known to prevent wound bleeding, nose bleeds, menstrual disorders, hematuria, hemoptysis, and bleeding hemorrhoids [22]. Moreover, this plant possesses anti-inflammatory [23], anti-oxidant [24], and anti-microbial [25] characteristics and has also been used to treat infection and rheumatism [26].

German chamomile, *Matricaria chamomilla* L., is an annual plant with a height of 40 to 20 cm. It has white flowers with yellow florets in the middle, yet its useful parts are the capitols which are separated from the stems from April to September. Chamomile has been a local plant of the Mediterranean [27]. In recent years, this plant has been used as an antioxidant [28], anti-cancer and pain-relief agent [29], and wound healer [30] due to its variety of biologic agents such as flavonoids and terpenoids [31]. Furthermore, this plant has been effective in the treatment of digestive tract spasms, anxiety, cholesterol, and blood pressure [32]; it is also used as a tranquilizer, immune booster, pain reliever, and wound healer in traditional medicine [33].

Turmeric (*Curcuma longa* L.) is a plant from the Zingiberaceae family. A herbaceous, perennial plant, its height is 1 – 1.5 m and has a tumorous rhizome that grows on aerial stems [34]. This plant is local to hot climates in Asia such as India, Pakistan, Indonesia, Southern China, as well as Africa and Southern America, and it does not grow in Iran. The curcumin in Turmeric has diverse characteristics like anti-tumor, cholesterol-lowering, immune booster, preventing cardiovascular disease, reduction in arthritis and rheumatism, and protecting against Alzheimer’s [35]. Turmeric is globally popular due to its appealing culinary, cosmetic, and medical applications. Various medical activities such as protection against light, skin protection, anti-asthma, blood sugar reduction, pain relief [36], anti-microbial [37], antioxidant [38], and anticancer [39] have been reported for it [40, 41]. Recently, turmeric powder has been used as a traditional medicine for the management of digestive system ailments, especially gallbladder and liver disease, diabetes, rheumatism, sinusitis, anorexia, rhinitis, and cough [42].

The objective of the present study is the green synthesis of nanoparticles using *M. chamomilla*, *A. wilhelmsii*, and *C. longa* species and to explore their effect on the treatment of skin disorders.

The nanoparticles synthesized in this manner have the potential to be utilized in health and medical industries due to the absence of dangerous chemical ingredients. Moreover, the results indicate that this method is simple and environmentally friendly and represents a good anti-microbial and anti-cancer performance.

## Materials and methods

### Plant selection and sampling

The three species of Turmeric (*C. longa*), Chamomile (*M. chamomilla*), and Yarrow (*A. wilhelmsii*) were chosen for the study of wound healing and skin diseases considering the use of plants in traditional medicine. Yarrow was procured from the southern areas of Isfahan (Semirom), located from 30º43’’ to 31º51’’ northern latitude and 51º17’’ to 51º57’’ eastern longitude. Chamomile was supplied from Khuzestan province which is located in 31º20’’ northern latitude and 48º40’’ eastern longitude. The turmeric was local Indian and was bought from an herbal apothecary. Permission for collection of plant materials obtained from the Agricultural Jahad Office and also the owner of the farm. The study is in compliance with relevant institutional, national, and international guidelines and legislation.The harvested specimens were transferred to the laboratory and exposed to free air in shade to dry. One sample of each whole plant was collected and pressed. The plant was identified and recorded at the herbarium of the University of Kashan. The plant was identified by Gianluigi Bacchetta and recorded with code number 1611, 1612, 1613..

### The preparation of extract and their yield

The plant materials were initially washed with deionized water to eliminate dust and insects and were dried in a room in shadow and the absence of light. To obtain the extract in the laboratory, firstly 50 g of each species was turned into powder by mortar and reached 500 cc by adding deionized water. *A. wilhelmsii* for 30 min at 45ºC, *C. longa* for 30 min at 90ºC, and *M. chamomilla* for 5 min at boiling temperature were placed on the hot plate stirrer. For compound extraction, 10 g of each plant was mixed with 10 g of another and brought to 200 cc with deionized water. Compound extracts were placed on a hot plate stirrer for 10 min at 100ºC .. Finally, the cooling and extraction were done at room temperature. After cooling, the extract of all three plants and the compound ones were filtered by filter paper at 4000 rpm, and the filtered extracts were kept in dark containers in the fridge for later analysis. To determine the yield of extract using the dry weight of the extract and the dry weight of the plant, it was calculated as a W/W proportion times 100, giving the yield in percentages [43].

### The evaluation of anti-oxidant properties of extract by DPPH assay

In this method, a solution was made in eight different concentrations. Firstly, the stock solution was prepared by weighing 25 mg of each weighed extract and being brought to volume in a 25 mL volumetric flask by adding methanol. After the preparation of the stock solution, the needed concentrations were prepared in eight 10 mL flasks. The first concentration is the stock solution itself, and the second (5 mL), third (8 mL), fourth (2.5 mL), fifth (1 mL), sixth (1 mL of the 3^rd^ concentration), seventh (1 mL of the 6^th^ concentration), ad eighth (1 mL of the 7^th^ concentration) concentrations being brought to volume in transparent 10mL volumetric flasks by adding methanol.

After the preparation of solutions with different concentrations, the DPPH solution was prepared by adding 4.7 mg of the DPPH powder to a dark 50 mL flask and bringing it to volume by adding methanol. After that, eight dark 5 mL flasks were placed adjacent to the eight transparent flasks, and 1 mL of the solution in the transparent flasks was added to its respective dark flask, from diluted ones to more concentrated ones. Finally, 1 mL of the prepared DPPH solution was added to the dark flasks and became homogenous. The dark flasks were kept at room temperature for half an hour. Eventually, the solution absorptions were determined by spectrophotometry. After half an hour the absorption of the prepared solutions was read in the wavelength of 517 nm in order from the control solution to the rest of the solutions from more diluted to more concentrated ones. After the calculation of the inhibition percentage by the concentration negative logarithm chart in Excel, the IC50 was duly calculated in µg/mL. All the steps were repeated three times.

The inhibition percentage is calculated by the formula below:

$$\mathrm{Inhibition\;percentage}= \frac{\mathrm{Control\;Absorption}-\mathrm{Sample\;Absorption}}{\mathrm{Control\;Absorption}} \times 100$$

### Silver nanoparticle synthesis

At this stage, the synthesis of nanoparticles was carried out in two methods. In the first method, 10 cc extract was mixed with 90 cc of 5mM silver nitrate solution; in the second method, 10 cc of extract was mixed with 90 cc of 5mM silver nitrate solution and 10 cc lye (NaOH). To prepare the lye, 0.04 g of dry lye was weighed by laboratory scale and reached 100 cc by adding deionized water. After the synthesis of nanoparticles, all extracts in the mentioned methods were kept at laboratory temperature for two hours.

To identify the nanoparticles and carry out further tests, the solution containing the nanoparticles was centrifuged for 15 min at 4000 rpm. The resulting solution was divided equally into test tubes, and the solution on top was separated slowly by a pipette. The resulting sediment was placed in the oven at 80 ºC for 24 h. After drying, it was powdered and kept in penicillin bottles at room temperature for further tests.

#### Identifying the synthesized nanosilver

##### Identifying nanoparticles by a spectrophotometer

In a UV-vis spectroscopy, light absorption causes electron excitement from the molecules and atoms’ valence shell which causes thin absorption lines in the case of atoms and broad and continuous absorption peaks in the case of molecules. Nanosilver particles exhibit a high capacity to absorb electromagnetic waves in the visible light spectrum, the maximum of which happens in the wavelength range of 420-450 nm due to a phenomenon called Surface plasmon resonance (SPR) [44]. For this test, after the synthesis of nanoparticles and changing their color to brown, the samples (with and without NaOH) were kept at lab temperature for 2 h, 48 h, 4 days, and 7 days in order, and studied by spectrophotometry in the range of 300 to 700 nm.

##### The study of the nanostructure using Scanning Electron Microscope (SEM)

The scanning electron microscope (SEM) is one of the strongest diagnostic tools to image the surface morphology and size distribution of nanosilver particles. To utilize SEM, the sample is covered by a thin layer of platinum. The electron is reflected from the sample layer in the SEM and collected by the detectors. These responses are then turned into light photons to make a visible image [45]. This test was carried out for all the prepared nanoparticles (with NaOH) and a compound sample from all three species (without NaOH) by the SEM.

##### Sample analysis by X-ray powder diffraction (XRD)

XRD is a valuable research tool used to verify the synthesis of nanoparticles, calculate the size of crystal particles, and determine the crystal structure [45]. When an X-ray is reflected on each crystal, various diffraction patterns are formed which indicate the physical and chemical characteristics of the crystal structure. Diffraction patterns usually show the physical and chemical structure of a powder sample. Thus, XRD can analyze the structural characteristics of a wide range of materials such as inorganic catalysts, superconductors, biomolecules, glasses, polymers, etc. [46].

In this method of analysis, all the synthesized nanoparticles (with and without NaOH) were analyzed in powder form.

##### Fourier Transform Infrared Spectroscopy (FTIR)

In this method, a spectrometer was used in order to investigate the possible organic compounds in the plant extract that are involved in the synthesis of nanoparticles. For this purpose, the dry extract and nanoparticles made in the form of pure powder were manually ground with a KBr ball and used for FTIR measurement. AgNP samples were separated through needle channels with 0.45 mm pores and sent for analysis. The retention spectrum was recorded between the frequencies of 400-4000 cm^-1^ [45].

### Anti-microbial activity

#### Microbe variants

In this test, clinical variants of gram-negative bacteria *Acinetobacter baumannii*, and gram-positive bacteria *Staphylococcus aureus* and *Staphylococcus epidermidis were* procured from the Iranian Research Organization for Science and Technology were used.

#### Agar well diffusion method

The Agar diffusion method was carried out as per CLSI standards [47]. In this method, plates containing Mueller–Hinton agar growth medium were prepared with 6 mm diameter wells on the medium. Then, 100.0 µL of bacterial suspensions with 0.5 McFarland turbidity were cultured in the growth medium. The dried extract was solved in Dimethyl sulfoxide and brought to 30.0 mg/mL,10.0 µL of which, equivalent to 300.0 µg was poured into each well. The plates were placed in an incubator for 24 h at 37 ºC. After that, the anti-microbial activity for each micro-organism was carried out by measuring the inhibition zone (in mm) with a sliding caliper. To evaluate the reproducibility and validity of the tests, each was repeated three times and the inhibition zone diameter was reported as mean ± standard deviation.

#### The measurement of Minimum Inhibitory Concentration (MIC)

This method was carried out by microdilution based on the MIC for micro-organisms susceptible to extract and silver nanoparticles. To each sterilized 96-well microplate, 95 µL growth medium, 5 µL bacterial suspension of 0.5 McFarland turbidity, and 100 µL of various concentrations of extract/nanosilver were added. After that, the plates were heated in an incubator for 24 h at 37 ºC. MIC was determined with regards to the turbidity and the color change spectrum in each microplate well. The test was repeated three times for each sample and the average minimum /nanosilver concentration with inhibitory properties against the bacteria or yeast was reported as MIC.

#### The measurement of minimum bactericidal concentration (MBC)

In this method of determining the MBC, after 24 h heating, the nutrient agar medium was inoculated with 5 µL of each microplate well that did not exhibit any growth and was heated for 24 h at 37 ºC. MBC is the lowest concentration of the extract in which %99.9 of the inoculated bacteria are killed.

### Statistical analysis

Statistical analysis of the extract yield was carried out in SPSS 22.0. Firstly, the normality of the statistical variables was assessed by the Kolmogorov–Smirnov test. After ascertaining the normality of the data, the ANOVA test was utilized to validate the statistical significance. The comparison of means was done by the Duncan test with a probability level of %1.

## Results and Discussion

### Extract yield

The findings from comparing the yields of extract samples showed that the highest yield belonged to the *M. chamomilla* species with a %11.8 mean, and the lowest was indicated by the compound extract (*C. longa + M. chamomilla* ) with a %1.055 mean. As is stated in the table, the mean yield of *M. chamomilla* species decreases when combined with other extracts. Moreover, *A. wilhelmsii* stood in second place with a mean yield of %7.66, and it also exhibited lower yields when combined with other extracts. The findings indicated that the *C. longa* species with a mean yield of %2.614, had a higher yield when combined with the other species (Table 1).

**Table 1:** Yield of pure and combined extract of studied species

extract	standard deviation ± mean
*A. wilhelmsii*	7.66 ^b^ ± 0.01
*M. chamomilla*	11.108 ^a^ ± 0.03
*C. longa*	2.614 ^e^ ± 0.00
*A. wilhelmsii+ M. chamomilla*	5.83 ^c ^± 0.03
*A. wilhelmsii+ C. longa*	3.435 ^d^ ± 0.03
*M. chamomilla+ C. longa*	1.055^ f ^± 0.02
*A. wilhelmsii+ M.chamomilla+ C. longa*	2.715 ^e^±0.02

Different letters in each column indicate significant differences based on Duncan's multiple range test at the 5% error level

One of the reasons for the difference in the extract, yields can be the effects of habitat on the quantity and concentration of secondary metabolites. The place of growth can impact the process of active ingredient formation via temperature and humidity variations. Furthermore, the relative rainfall of the area and the organic matter in the plant, the temperature (one of the ecological factors limiting the plant growth and the synthesis of active ingredients in herbal medicine), and ecological factors are the effective factors in the extract yield. Another effective factor in the extract yield is the plant species [48]. The present results agree with the results reached by [49] on the *Thymus vulgaris* L. extract and [50] on the *Zingiber Officinale* L. species.

The change in the color of the extract solution after adding the silver nitrate solution is because of the reduction of metal ions in the reaction. The darkening in the color of the solution in time is also related to the surface plasmon resonance of silver nanoparticles (AgNp_s_) and verifies their synthesis [51].

**Fig. 1:**
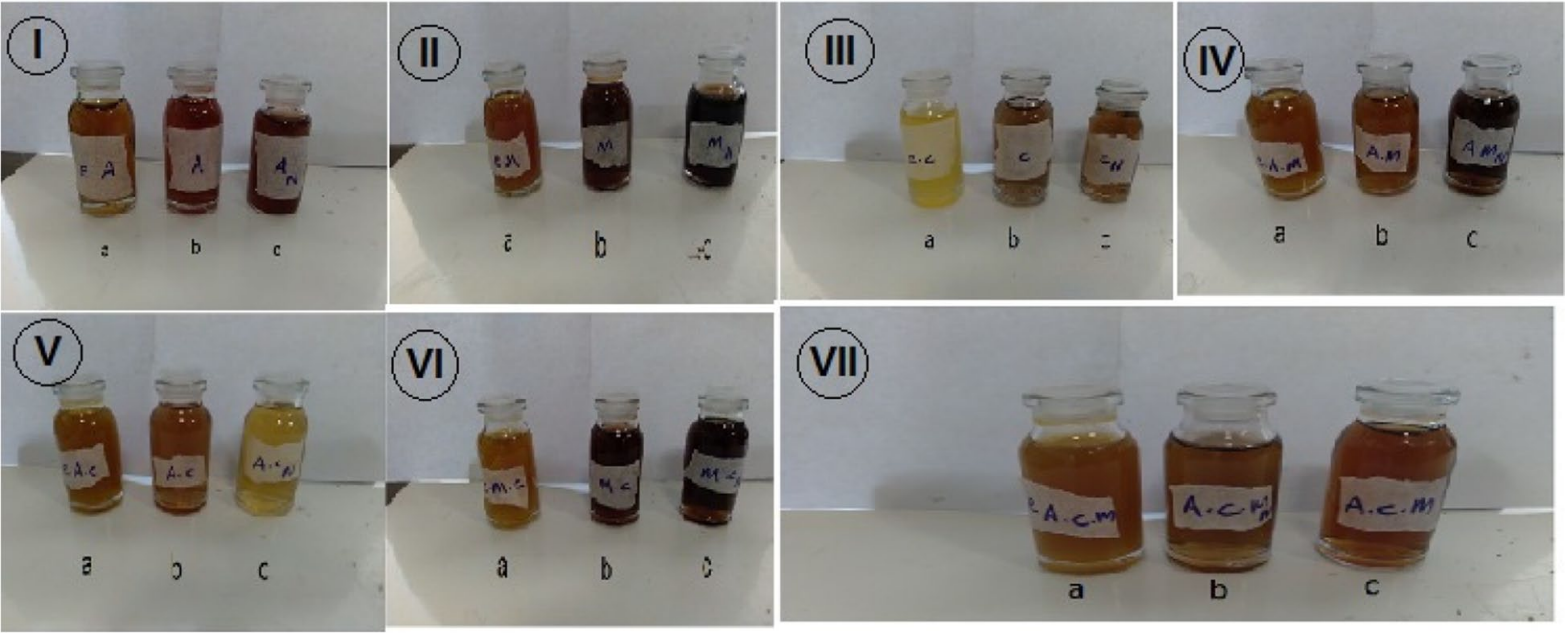
Color change of silver nanoparticle synthesis. a=Extract, b= AgNPs, c= AgNps+NaOH. *A.wilhelmsii* )I), *M. chamomilla*) II)*, C. longa* (III), *A. wilhelmsii+ M. chamomilla* )IV), *A. wilhelmsii+ C. longa* (V), *M. chamomilla+ C. longa*) VI)*, A. wilhelmsii+ M. chamomilla+ C. longa *(VII)

After adding silver nitrate to the extracts and the synthesis of silver nanoparticles, with the passing of tie, the color of the solutions changed from light yellow to brown (Fig. 1).

As per the observations made, after the synthesis of nanoparticles and the addition of NaOH to the synthesized nanoparticles, the resulting solution which was mostly yellow, changed to dark brown which became darker as time passed. The change in the nanosilver solution color is a variable of time, which agrees with the research results of [52] on *A.wilhelmsii* and [53] on *Fumaria officinalis* L.

#### Nanoparticle analysis with UV-vis

UV-vis spectroscopy is a useful and reliable technique for the primary detection of synthesized nanoparticles. It is also used to supervise the synthesis and stability of silver nanoparticles. Moreover, it facilitates fast, easy, simple, sensitive, and selective spectrometry for various nanoparticles, as well as a short period for measurement and no need for calibration to determine the characteristics of colloid suspension particles [46]. To identify the peak absorbance of the samples in a defined range, the wavelength with the highest absorbance was chosen as the sample peak absorbance [54].

In this study, the 300-700 nm wavelength was chosen for the evaluation of silver nanoparticles for the duration of 2h, 48 h, 4 days, and 7 days.

**Fig. 2:**
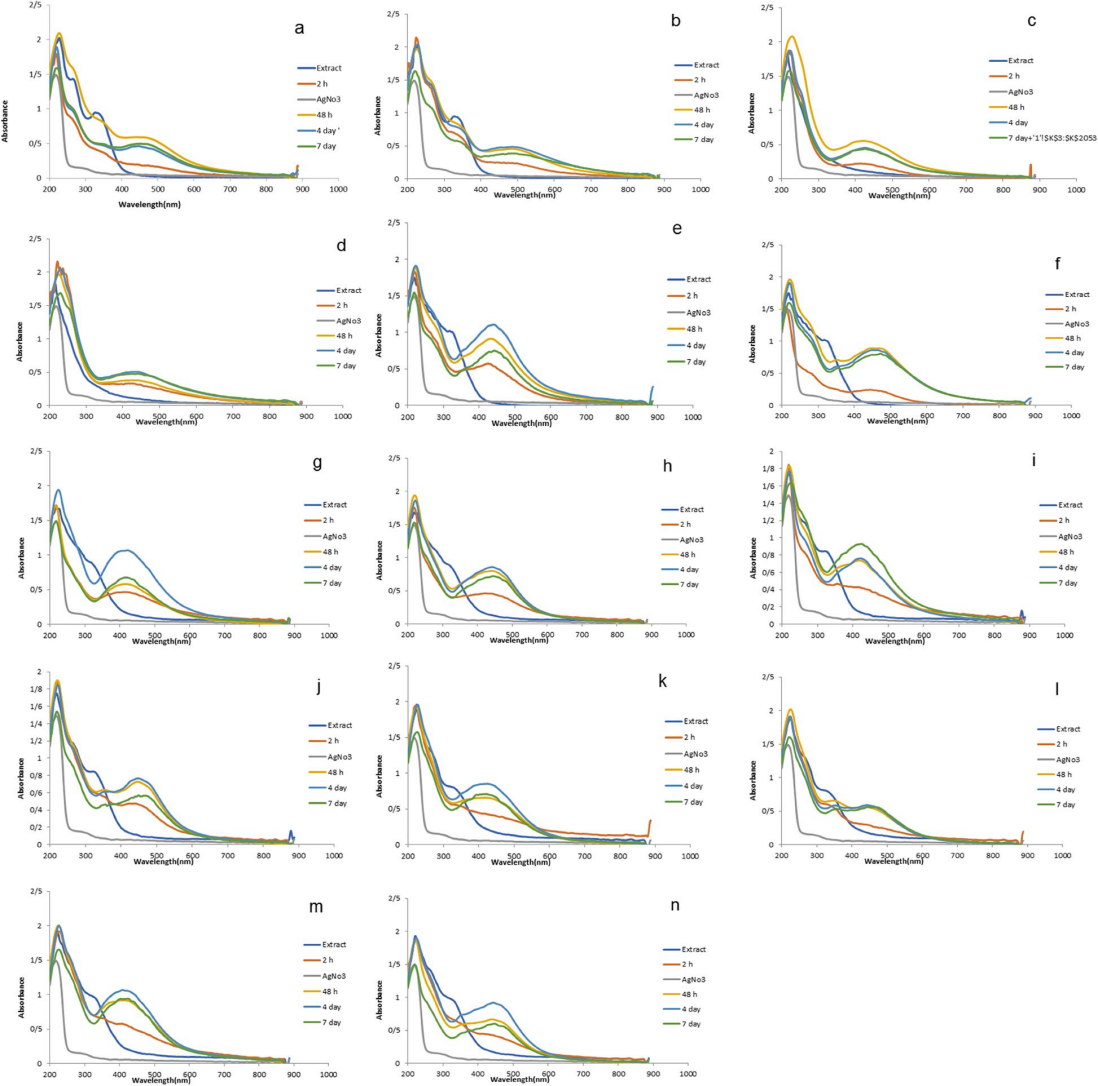
Absorption spectra of silver nanoparticles and study of the effect of time on the sample adsorption intensity: *A. wilhelmsii+*NaOH (**a**), *A. wilhelmsii* (**b**), *M. chamomilla+* NaOH (**c**), *M. chamomilla *(**d**)*, C. longa+* NaOH (**e**), *C. longa *(**f**), A. wilhelmsii+ M. chamomilla+NaOH (**j**), *A. wilhelmsii+ M. chamomilla* (**h**) .(*A. wilhelmsii+ C. longa+* NaOH (**i**), *A. wilhelmsii+ C. longa* (**j**), *M. chamomilla+ C. longa+* NaOH (**k**), *M. chamomilla+ C. longa*) (**l**), *A. wilhelmsii+ M. chamomilla+ C. longa+* NaOH (**m**), *A. wilhelmsii+ M. chamomilla+ C. longa *(**n**)

As per the results of the (*A. wilhelmsii+*NaOH) absorption spectra after 2h, 48 h, 4 days, and 7 days, absorbance peaks of 403, 443, 459, and 451 nm were observed respectively (Fig. 2 (a and b)). The synthesized samples of this extract which did not contain NaOH formed peaks in the range of 403, 482, 486, and 486 nm respectively. The absorption spectra obtained from the other samples formed peaks in the 400-500 nm range, like the *A. wilhelmsii+*NaOH species (Fig. 2 (c to n)).

As can be seen in all the figures, the absorption spectra of silver salt do not exhibit any significant peak absorbance in the 300-700 nm range.

The findings showed that after mixing the silver salt in an extract solution and performing analysis in the different durations of 2 h, 48 h, 4 days, and 7 days, peak absorbances were formed between 400 and 500 nm which indicates the biosynthesis of silver nanoparticles. The existence of a peak in the wavelength of 400 to 450 nm shows the synthesis of silver nanoparticles and is related to the surface plasmon resonance of silver nanoparticles which is attributed to the induction of free electrons in nanoparticles [54]. Each of the samples formed a peak absorbance in a specific area between 400 and 500 nm, a result which is in accordance with the research results of [9] on *C. longa*, [55] on *M. chamomille*, [56] on *Organnum Majorana .*L, [57] on *Salvia officinalis.* L.

One of the most important factors in the biosynthesis of silver nanoparticles is the effect of time because the redox process for the compounds in the extract causes a reduction in the silver salt and turns it into silver nanoparticles. Therefore, for the completion of this process and complete reduction of the silver salt and its transformation to nanoparticles, too little time will disrupt the possibility of the synthesis of all the silver ions into nanoparticles, and too much time will not have a significant effect. Thus, it is necessary to optimize the time to add speed and allow a full synthesis of nanoparticles [54].

According to the results achieved from the UV-vis spectrometry, the silver absorption increased by increasing the time from 2 h to 48 h, a fact that is related to the rise in the concentration of synthesized silver nanoparticles. However, most samples exhibited the same absorption and little change after 4 and 7 days. Thus, 48 h seems to be an ample time for the synthesis of silver nanoparticles from silver ions, and increasing the time does not have a significant effect. Our results agreed with the research results of [58] on *A. wilhelmsii,* [59] on *A. wilhelmsii,* [52] on *A. wilhelmsii,* and [60] on *Syzygium aromaticum* (L.) Merr. & L.M. Perry.

To verify the explanations about the time factor, the changes in the nanoparticle synthesis in 2 h, 48 h, 4 days, and 7 days were observed, which indicated that the solution got darker from 2 h to 48 h, but did not exhibit any significant changes after that. This further verifies the findings of this method and the optimal time determined. Another factor studied and analyzed in this test was the effect of NaOH on the biosynthesis of nanoparticles [52]. After the tests, the absorption charts indicated that adding NaOH did not have a significant impact on the samples, especially the compound ones. The only difference was observed when the peak absorbance of some samples such as non-compound ones had a slight increase compared to the samples containing NaOH when the time was risen. Therefore, a time of 48 hours and not using NaOH is suggested for synthesis.

#### The analysis of nanoparticles by X-ray Diffraction (XRD) analysis

X-ray diffraction (XRD) is a popular analytical technique for analyzing molecular and crystal structures, qualitative identification of various compounds, quantitative separation of chemical types, the measurement of crystallinity index, isomorphous substitutions, and the size and purity of particles, etc. The analysis of these materials depends greatly on the formation of diffraction patterns. Each material has a unique diffraction beam which can be compared and identified using the diffused beams in the reference database of the International Centre for Diffraction Data (ICDD). Diffraction patterns also indicate the purity or impurity of the samples. Therefore, XRD has been used for the definition and identification of bulk and nanomaterials, forensic samples, and industrial and geochemical samples [46].

The X-ray diffraction pattern for all samples (wit and without NaOH) with 2ϴ equaled 38.20, 44.37, 64.57, and 77.48 which respectively corresponded to the crystal plates (111), (200), (220), and (311) and indicate the crystal nature and an FCC cubic structure. The miller indices of these peaks compared with standard card JCPDS number 4-0783 [61].

Moreover, some sharp high intensity peaks in the XRD patterns show that they are mainly composed of AgCl. The main peaks in 2ϴ equaled 27.9, 32.3, 54.9, 57.5, 67.2, 74.6, and 76.8 which were attributed to cubic crystallography plates (111), (200), (220), (311), (222), (400), and (331) respectively (JCPDS number 31-1238) [62].

**Fig. 3:**
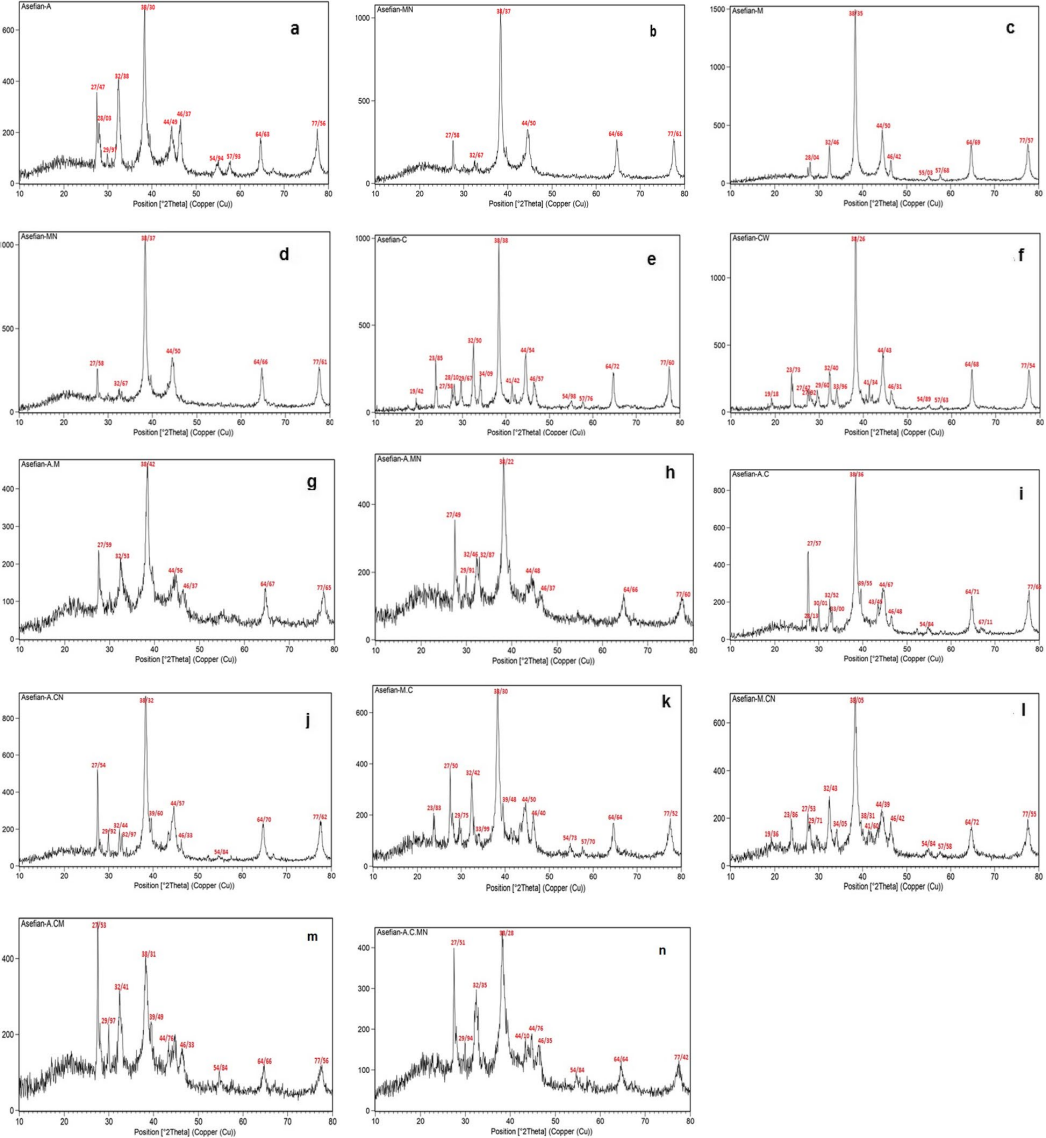
XRD spectrum image of silver nanoparticles. *A. wilhelmsii+*NaOH (**a**) ( *, A. wilhelmsii* (**b**), *M. chamomilla+* NaOH (**c**), *M. chamomilla *(**d**)*, C. longa+* NaOH (**e**), *C. longa *(**f**), *A. wilhelmsii+ M. chamomilla+*NaOH (**j**), *A. wilhelmsii+ M. chamomilla* (**h**) .(*A. wilhelmsii+ C. longa+* NaOH (**i**), *A. wilhelmsii+ C. longa* (**j**), *M. chamomilla+ C. longa+* NaOH (**k**), *M. chamomilla+ C. longa*) (**l**), *A. wilhelmsii+ M. chamomilla+ C. longa+* NaOH (**m**), *A. wilhelmsii+ M. chamomilla+ C. longa *(**n**)

For instance, the XRD results for the (*A. wilhelmsii* + NaOH) synthesized nanoparticles indicated 11 main peaks which, as per the comparisons carried out, peaks 5, 6, 10, and 11 were related to silver and peaks 1, 4, 8, and 9 were related to silver chloride (Fig. 3).

The spectrum resulting from the silver nanoparticles in other samples also shows several peaks in samples, some of which are related to silver and some others to silver chloride.

The mean particle crystallization was calculated implementing the Debye Scherrer equation in which L represents the size of the particles in nm, θ is the Bragg angle in rad., λ is the X-ray source wavelength (1.5406), β is the full width at half maximum, and K is the Scherer's constant (0.94) [61].

$${L}=\frac{{k}{\gamma}}{{\beta}{cos}\mathbf{\infty }}$$

As per the calculations carried out in Table 2, the mean particle size in samples with and without NaOH was measured using the equation above. The XRD results indicated the presence of pure silver, as separate strong peaks, in samples synthesized from the extract of all three plants (with and without NaOH) in crystal plates 111, 200, 220, and 311. Our results agreed with the results of numerous researchers such as [63] on *Z*. *officinael,* [9] on *C. longa,* [59] on *A. wilhelmsii,* [51] on *Alpinia nigra* B.L, [64] on *M. chamomilla,* [52] on *A. wilhelmsii,* [56] on *O. Majorana* L, and [65] on *Rheum ribes.* The study on the silver nanoparticles synthesized from the designated extracts indicated the presence of other elements besides pure silver, which combine with silver and cause the existence of other compounds in the synthesized samples. In these tests, other than the pure silver present in the extracts, chlorine was also detected which can be due to its high amount in the extracts and was represented in combination with the silver as silver chloride (AgCl). Therefore, the samples had crystal plates (111), (200), (220), (311), (222), (400), and (331). The samples without lye showed more silver chloride than the samples containing NaOH. The size of the particles in the samples containing NaOH was slightly less than the samples without NaOH, the most significant of which was related to the compound sample *A. wilhelmsii+ M. chamomilla.*

**Table 2:** The measurement of silver nanoparticles (NaOH) by XRD method

Sample	β	$\infty$2	L(nm)
*A. wilhelmsii*	0.2952	38.3123	4.91
*M. chamomilla*	0.2952	38.3501	4.91
*C. longa*	0.2362	38.3876	4.95
*A. wilhelmsii+ M. chamomilla*	0.5904	38.4257	6.25
*A. wilhelmsii+ C. longa*	0.5314	38.3656	3.23
*M. chamomilla+ C. longa*	0.5314	38.3031	3.23
*A. wilhelmsii+ M. chamomilla+ C. longa*	0.2362	27.9714	2.85

#### Analysis of nanoparticles implementing Fourier Transform Infrared Spectroscopy (FTIR)

Fourier Transform Infrared Spectroscopy (FTIR) is used to detect biomolecules that have reducing or screening roles in the Ag reduction. Thus, IR spectroscopy is a suitable method for the detection of bioactive components in natural products. Moreover, this technique is a valuable tool to identify the presence of secondary metabolites on silver nanoparticles in plants [44].

Furthermore, FTIR is developed to study materials on nanoscale such as verifying functional molecules in Ag covalent bonds, carbon nanotubes, graphene and gold nanoparticles, and interactions between enzymes and catalyst layers. Thus, FTIR is a suitable, valuable, non-invasive, affordable, and simple technique to identify the role of biomolecules in the reduction of silver nitrate to silver [46]. Retention spectra were recorded between 4000 cm^-1^ to 400 cm^-1^ wavelengths [66].

**Fig. 4:**
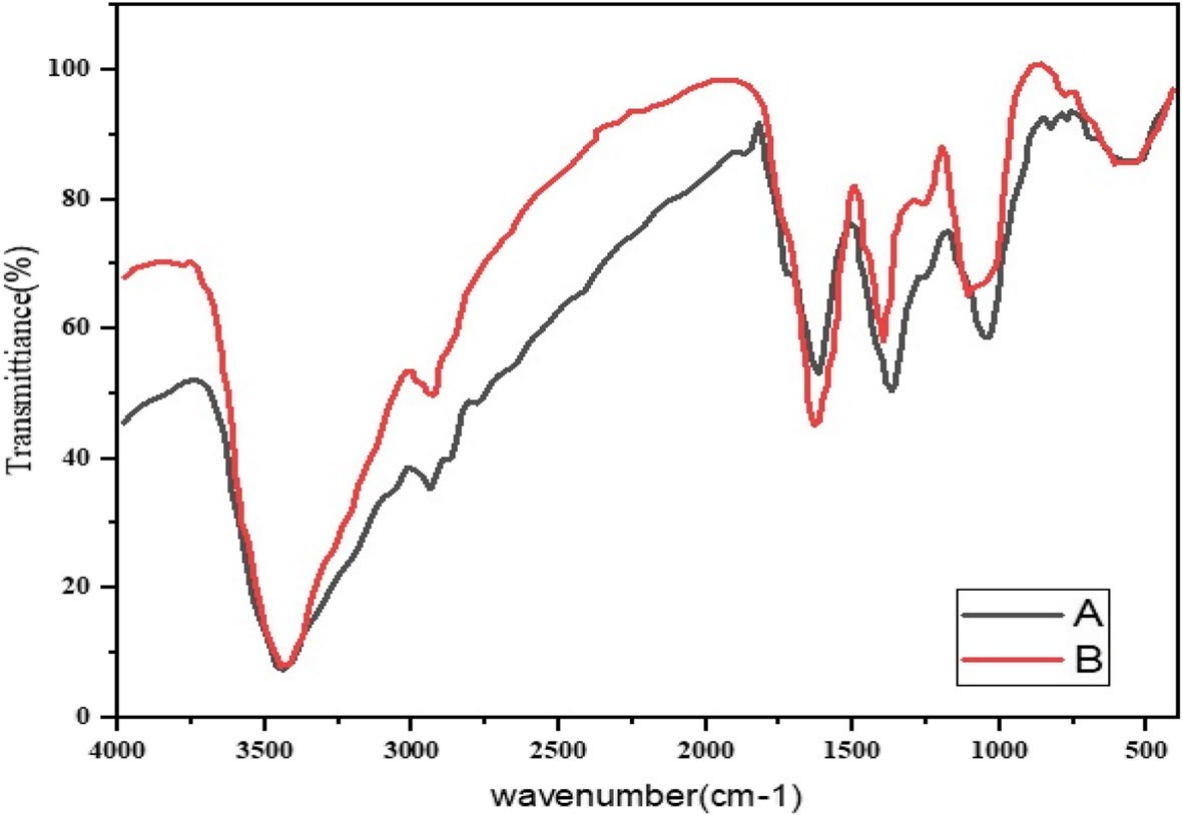
Infrared Fourier Transform Spectrometer (FTIR) Chart. A-*A*. *wilhelmsii* + NaOH (AgNPs), B- *A*. *wilhelmsii* (extract)

**Fig. 5:**
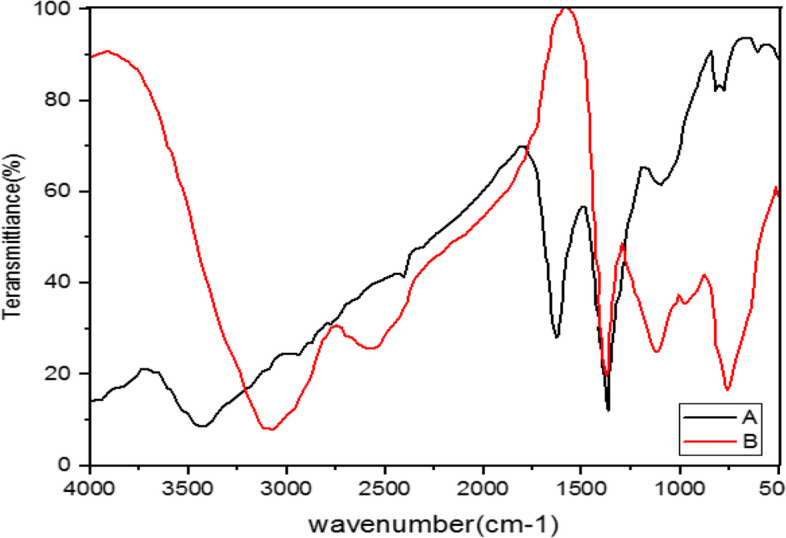
Infrared Fourier Transform Spectrometer (FTIR) Chart. A-*M.chamomilla* + NaOH (AgNPs), B- *M.chamomilla* (extract)

**Fig. 6:**
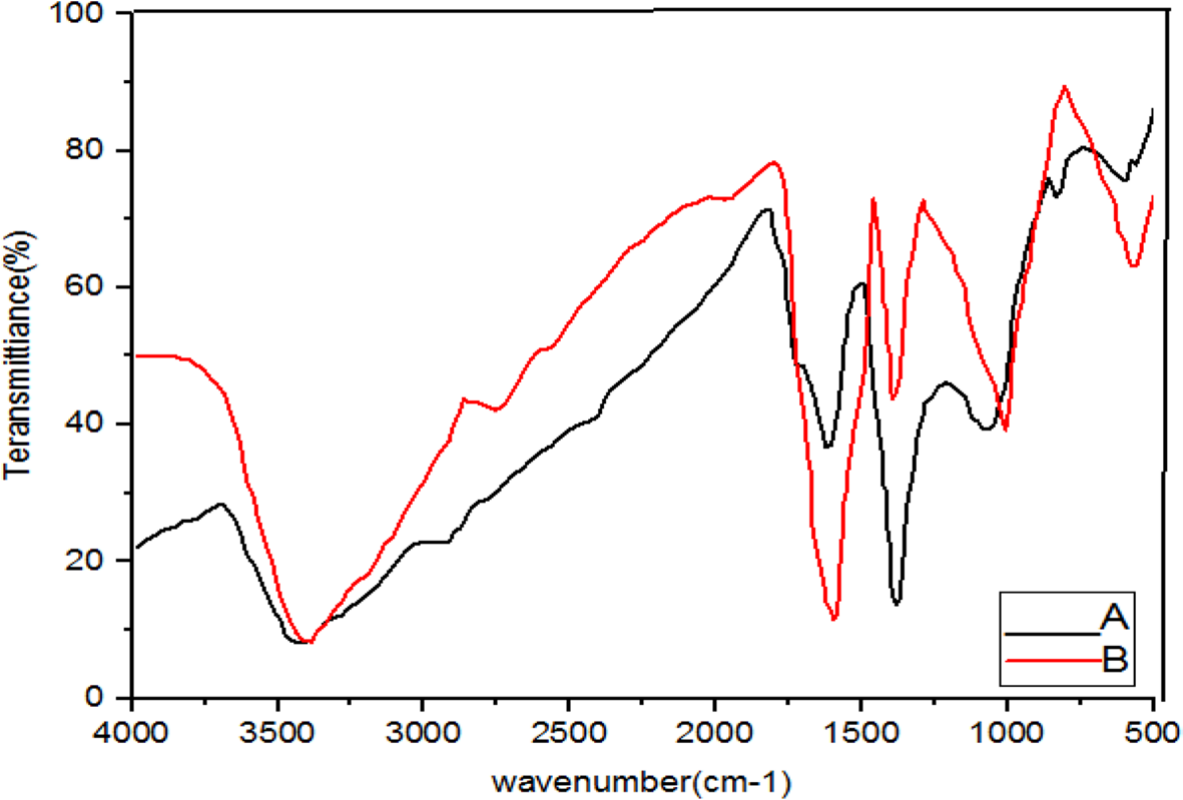
Infrared Fourier Transform Spectrometer (FTIR) Chart. A-*C*. *longa* + NaOH (AgNPs), B- *C*. *longa* (extract)

**Fig. 7:**
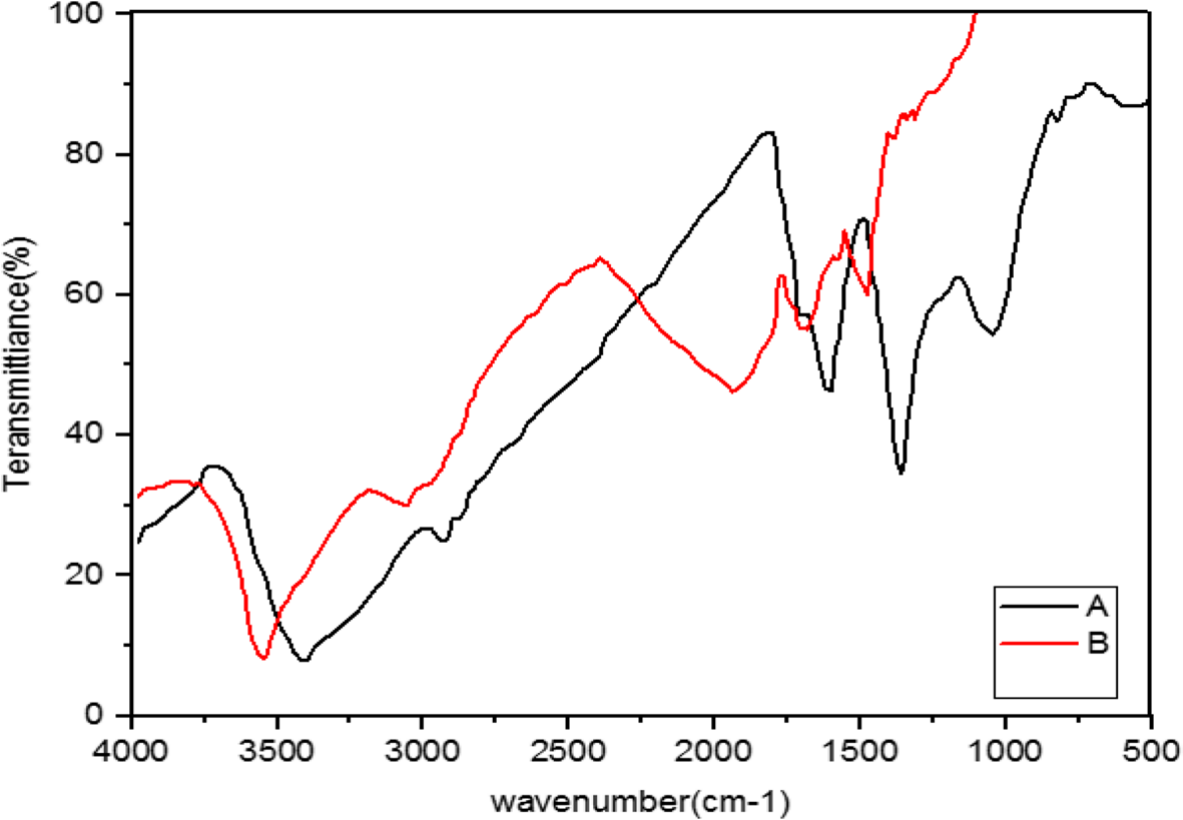
Infrared Fourier Transform Spectrometer (FTIR) Chart. A-*A*. *wilhelmsii* + *M*. *chamomilla* + NaOH (AgNPs), B- *A*. *wilhelmsii* + *M*. *chamomilla* (extract)

**Fig. 8:**
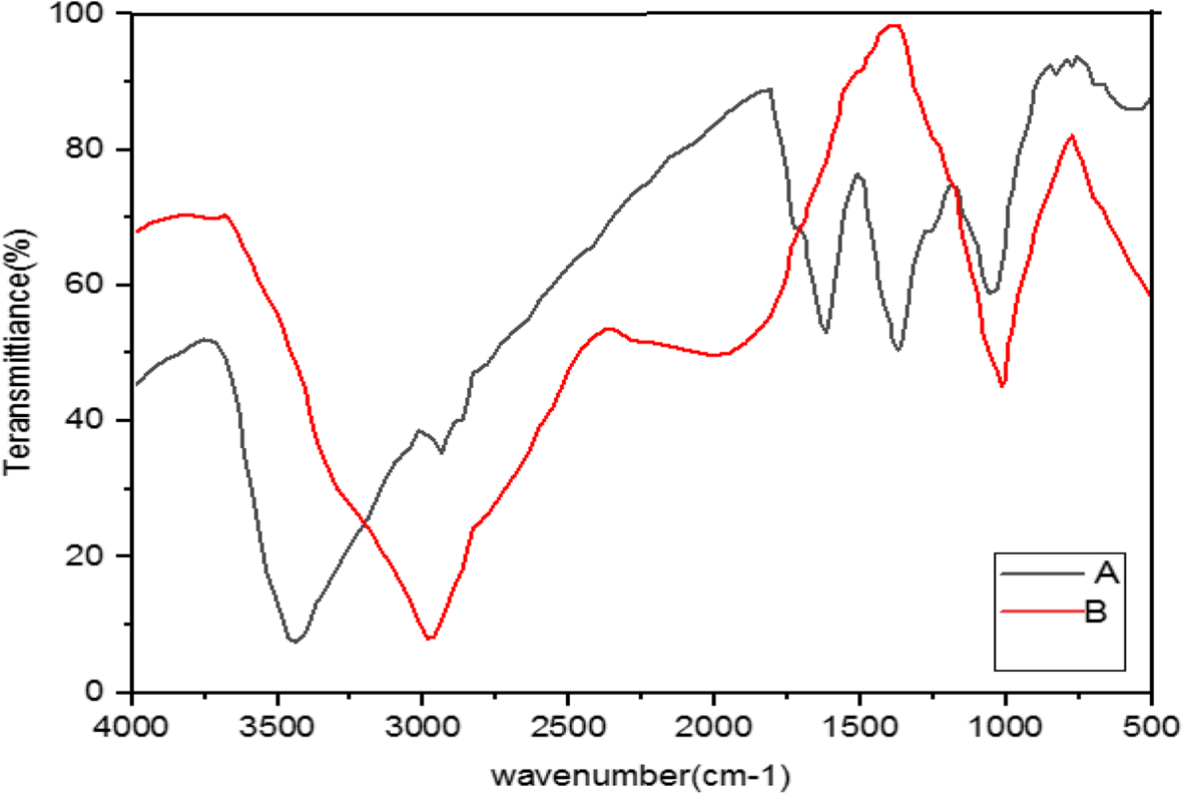
Infrared Fourier Transform Spectrometer (FTIR) Chart. ***A-****A*. *wilhelmsii* + *C*. *longa* + NaOH (AgNPs), B- *A*. *wilhelmsii* + *C*. *longa* (extract)

**Fig. 9:**
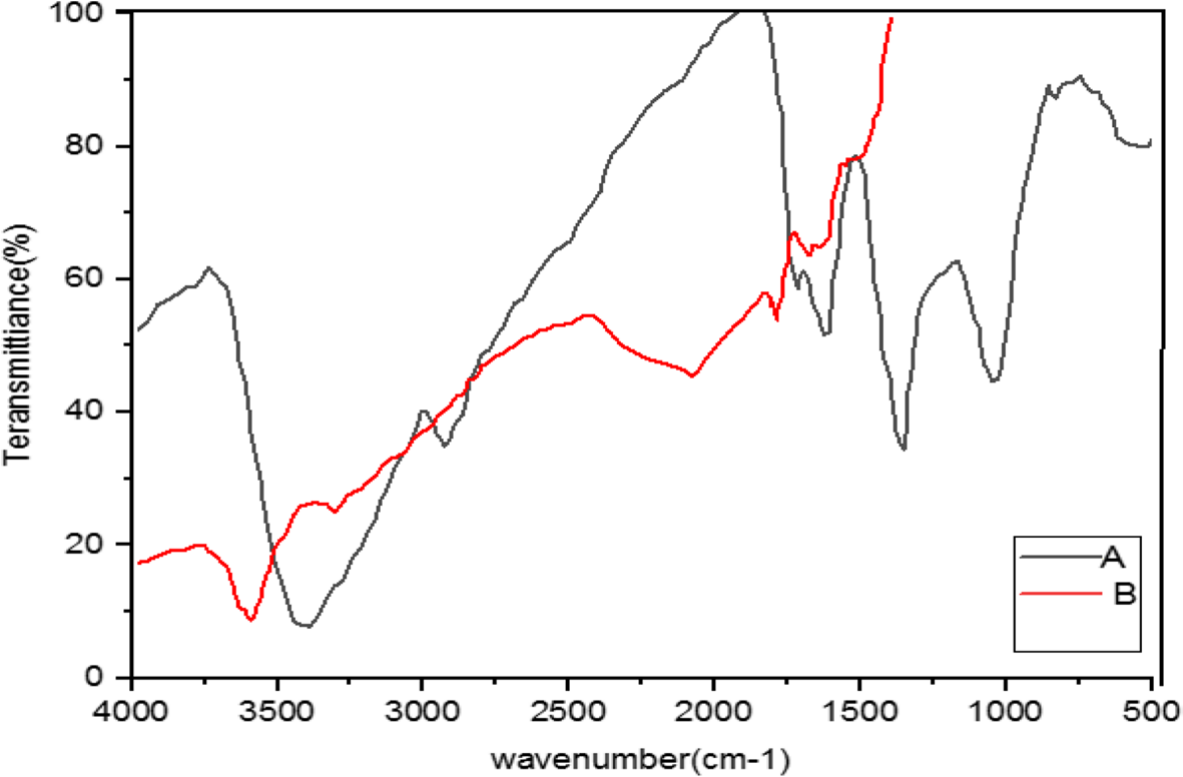
Infrared Fourier Transform Spectrometer (FTIR) Chart. A- *M*. *chamomilla* + *C*. *langa* + NaOH (AgNPs), B- *M*. *chamomilla* + *C*. *langa* (extract)

**Fig. 10:**
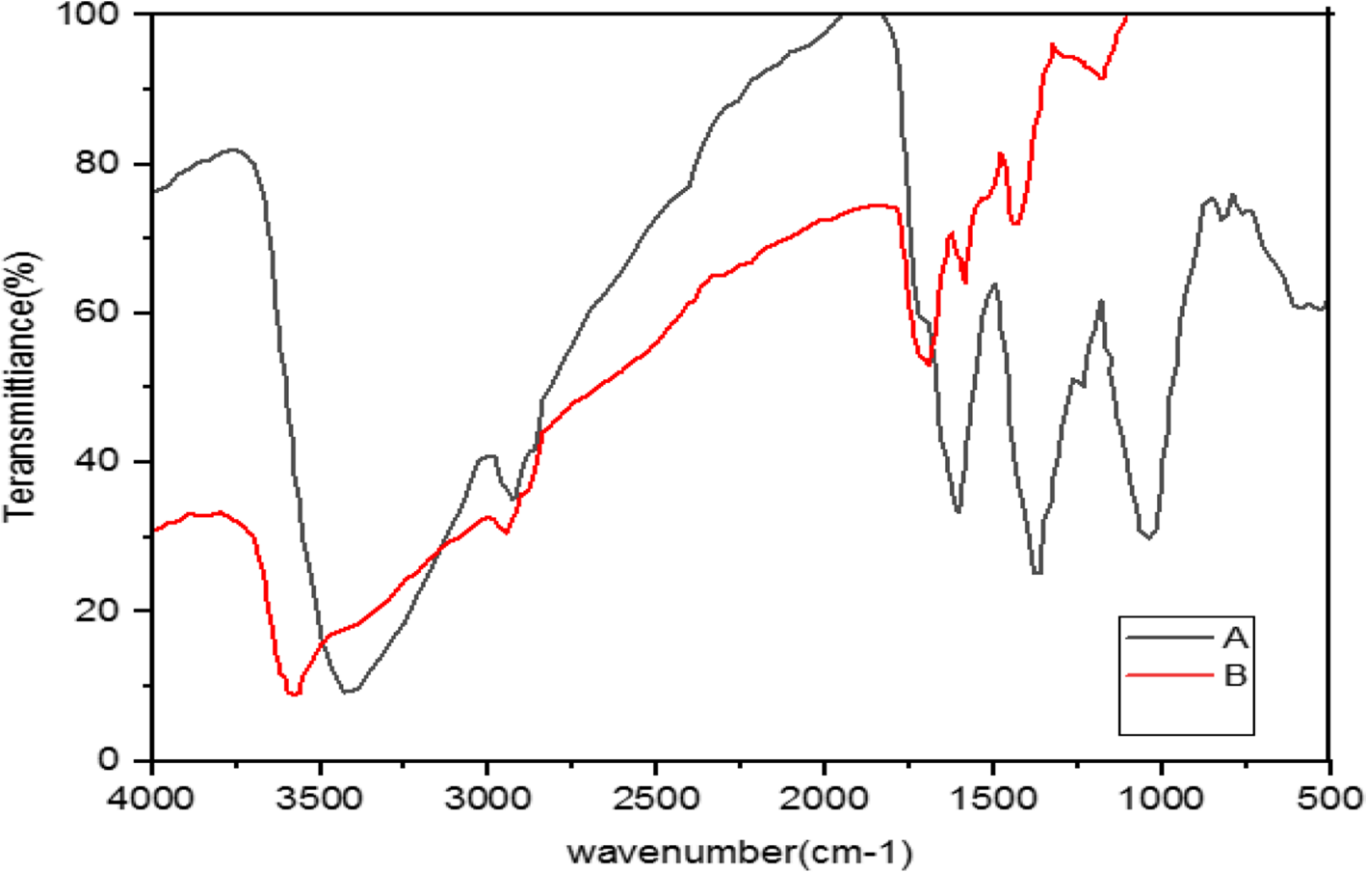
Infrared Fourier Transform Spectrometer (FTIR) Chart. A-*A. wilhelmsii* + *M. chamomilla* + *C. longa* + NaOH (AgNPs), B- *A. wilhelmsii* + *M. chamomilla* + *C. longa* (extract)

The FTIR spectrum is illustrated to study the interaction between silver nanoparticles and the extract of *M. chamomilla*, *A. wilhelmsii,* and *C. longa* before and after the synthesis of silver nanoparticles (Figs. 4, 5, 6, 7, 8, 9 and 10).

The FTIR results from the dried extract of *A. wilhelmsii* illustrated this spectrum between the absorption bands 3405.12 and 529.35 cm^-1^. Moreover, the main absorption peaks were shown at 2928.20, 1619.73, 1406.02, 1261.51, and 1064.92 cm^-1^. These peaks illustrate that various active groups such as alcohols, carboxylic acids, alkenes, alkanes, alkyls present in the solution have screening and stabilizing functions [67].

Regarding the *A. wilhelmsii* extract, a strong absorption peak was illustrated at 3405.12 cm^-1^, related to the active group hydroxyl (OH) with stretching vibrations (alcohol and phenol group) responsible for the reduction of Ag^+^ to Ag. Therefore, it can be said that the biosynthesized silver nanoparticles were stabilized by the polyphenol compounds of the extracts, which confirms the antioxidant activity of the extracts [68]. The bending vibration of the OH group was detected at the peak of 1619.73 cm^-1^. The absorption peaks were illustrated in bands 1406.02, 1261.51, and 1064.92 cm^-1^ which were respectively related to the active C-H group with bending vibration (alkanes), C-O (esters, ethers, carboxylic acids, and alcohols), and C-O [69] showed similar FTIR manner after AgNPs synthesis by alginate.

The spectrum of nanoparticles from *A. wilhemsii* extract illustrated a broad and strong peak in the band 3423.14 cm^-1^ which indicates stretching vibrations of OH in phenol and alcohol compounds. The distinct peak at 1634.73 cm^-1^ indicates OH bending vibrations of alcohol groups. Moreover, new absorption peaks at 1383.95, and 1042.10 cm^-1^ were attributed to active C-O groups.

The extracts of *M. chamomilla* and *C. longa* also illustrated peaks similar to the active groups of the *A. wilhemsii* extract, although *C. langa* extract exhibited 1595.19 and 1122.41 cm^-1^ peaks of the active OH group with bending vibrations and C-O; the spectra from these extracts also exhibited similar active groups.

The FTIR results of the four compound extract samples illustrated similar peaks and equal active groups; moreover, the spectra from the synthesized nanoparticles also illustrated similar active groups same as the spectra from the single extracts.

The FTIR results illustrated that the extract molecules remained on the layer of synthesized nanoparticles and acted as screening and stabilizing agents [67]. The FTIR spectrum of silver nanoparticles synthesized from extracts is similar to the corresponding extract with some minor variations in the location of the bands. The extract peaks have a screening function; therefore, band locations are slightly moved; moreover, as illustrated in the figures, after reactions with silver nitrate there are some changes in the height and location of some peaks which is related to the breaking of chemical bonds in hydroxyl, carbonyl, and CO_2_ groups and the release of hydrogen and carbon which have roles in the reduction of silver ions [60].

The study of nanoparticles indicated the similarity between various groups of chemical agents. FTIR illustrated the repetition of stretching vibrations of the metal-oxygen bonds [66].

Strong and broad peaks illustrated in the FTIR chromatography indicate the presence of OH groups and were shown in all the samples, a fact that demonstrates the strong affinity of extracts towards layers of AgNPs, thus stabilizing the biosynthesised silver nanoparticles [44].

The FTIR results showed that some of the extract active biocompounds such as proteins and phenol compounds formed a strong careening on the resulting biosynthesis [45]. In addition, the observed peaks are more characteristic of flavonoids and terpenoids that are present in the studied plants. It can be speculated that these secondary metabolites are responsible for the synthesis/stabilization of silver nanoparticles [70]. Flavonoids are plant bioactive compounds that are of great interest in nutrition and pharmacology due to their remarkable properties as antioxidants, anti-inflammatory, antibacterial, antifungal and anti-tumor drugs [71]

#### Nanoparticle analysis by scanning electron microscope (SEM)

Among the various techniques of the electron microscope, SEM is a layer imaging method that is completely capable of identifying various sizes of particles, size distribution, the shape of nanomaterial, and the layer morphology of synthesized particles on micro and nanoscale.

Utilizing the SEM facilitates the study of the morphology of particles and extracting the histogram by measuring and counting the particles either by hand or using relevant software.

**Fig. 11:**
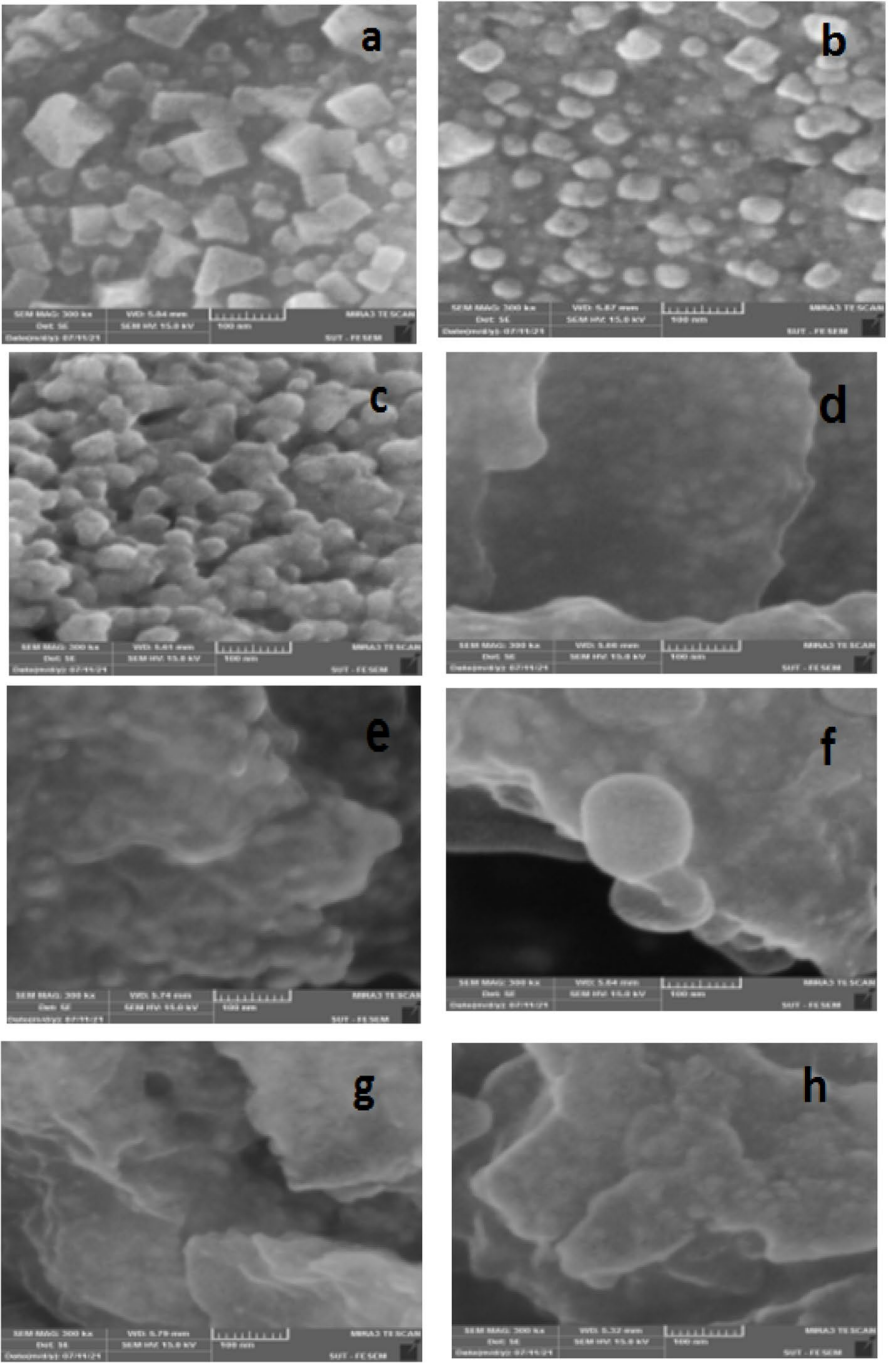
100 nm electron microscope (SEM) images of synthesized nanoparticles. Produced by species : *A. wilhelmsii+* NaOH) (**a**), (*C. longa+* NaOH) (**b**)*, M. chamomilla+* NaOH) (**c**)*, M. chamomilla+ C. longa+* NaOH (**d**)* , M. chamomilla+ A. wilhelmsii+* NaOH (**e**)*, A. wilhelmsii+ C*. *longa+* NaOH (**f**)*, A. wilhelmsii+ M. chamomilla+ C. longa+* NaOH) (**g**)*,* (*A. wilhelmsii+ M. chamomilla+ C. longa *(**h**)

The combination of SEM and Energy-dispersive X-ray spectroscopy (EDX) can be utilized to study the morphology of silver powder and the analysis of its chemical compound. SEM is limited in the study of the inside structure but can provide valuable information about the purity and degree of accumulation of the particles. The modern SEM can detect the morphology of nanoparticles under 10 nm with a high resolution [46]. The SEM results illustrated a mostly cubic morphology for the (NaOH*+A. wilhemsii*) species and the size of nanoparticles was between 20 and 80 nm. Furthermore, the synthesized nanoparticles in the (*C. longa+* NaOH) sample were spherical and their a size between 10 and 40 nm. The (*M. chamomilla+* NaOH) sample exhibited spherical particles adjacent to each other with a size between 10 and 30 nm. The compound sample of the species (*C. longa +A. wilhemsi+*NaOH) illustrated oval particles and a size of 50 nm approximately. The (*A.wilhemsi+ M. chamomill+*NaOH) sample illustrated spherical particles with the approximate size of 20 nm, and the (*C. longa + M. chamomill+*NaOH) illustrated nanoparticles of angular spherical Shape with a size of 10 to 20 nm. Moreover, the three compound species (*C. longa+A. wilhemsi+M. chamomilla+*NaOH) had spherical rod shapes adjacent to each other with a size of about 10 nm; and the three compound species (*C. longa+A.wilhemsi+M. chamomilla*) exhibited angular spherical nanoparticles with the size of 10 nm (Fig. 11)

The EDX analysis presents extra chemical data about the samples such as the elements and concentration [66].

**Fig. 12:**
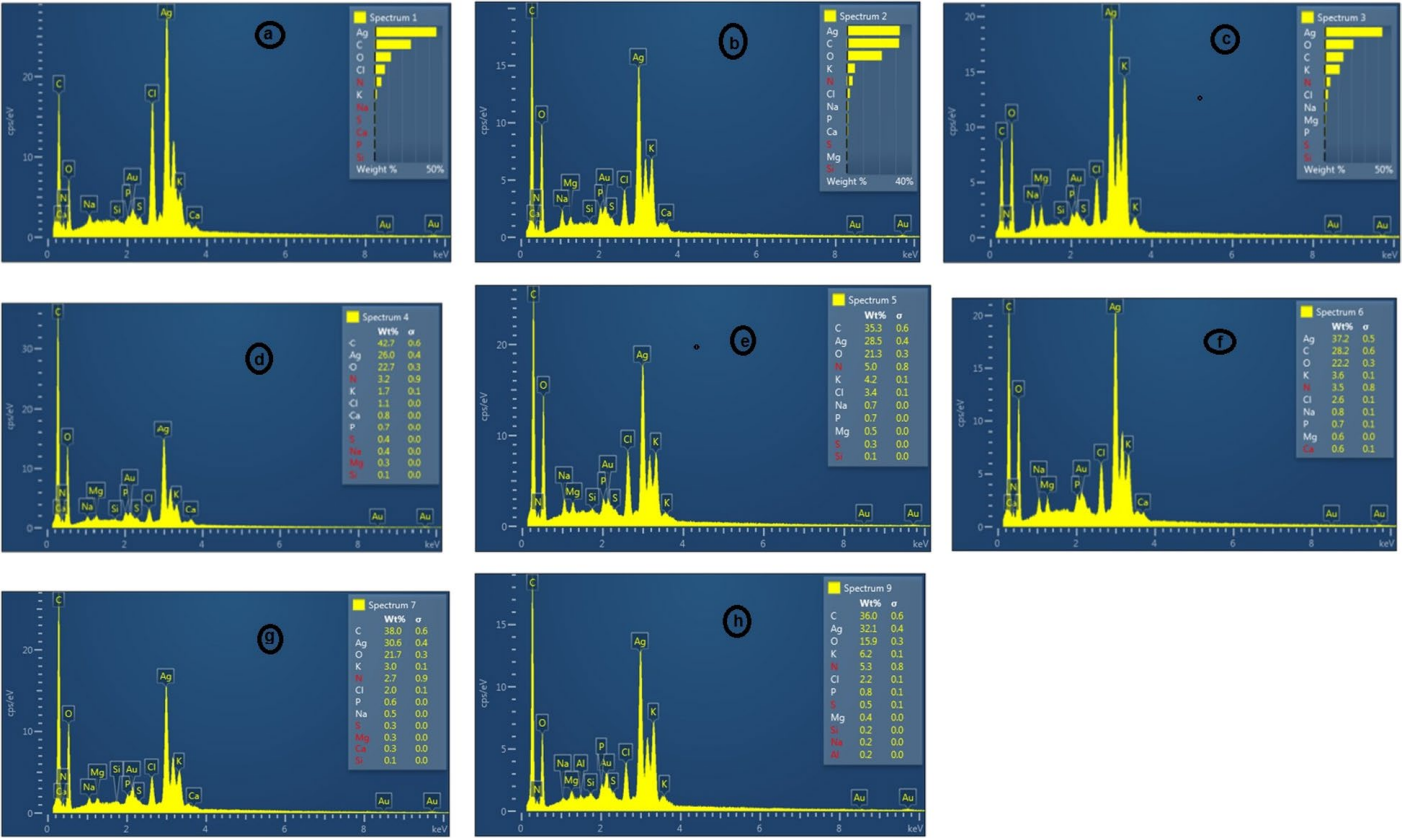
Image of X-ray scattering energy spectrum (EDX) of silver nanoparticles. *A. wilhelmsii+* NaOH) (**a**), (*C. longa+* NaOH) (**b**)*, M. chamomilla+* NaOH) (**c**) *, M. chamomilla+ C. longa+* NaOH (**d**)*, M. chamomilla+ A. wilhelmsii+* NaOH (**e**)*, A. wilhelmsii+ C*. *longa+* NaOH (**f**)*, A. wilhelmsii+ M. chamomilla+ C. longa+* NaOH) (**g**)*,* (*A. wilhelmsii+ M. chamomilla+ C. longa *(**h**)

EDX analysis illustrated a strong silver signal in the (*A*. *wilhemsii*+ NaOH) sample at 3 KeV, validating the presence of silver nanoparticles, with the silver weight percent of 45.47. In addition, other elements such as N (% 4.95), Cl (% 7.55), and O (% 11.98) were detected in this sample (Fig. 12).

In the (*C. longa* ) sample, a strong silver signal with a weight percentage of % 32.59 verified the presence of silver nanoparticles and other elements such as K (% 4.95), O (% 21.53), and C (% 32.15).

Likewise, a strong signal at 3 KeV in the (*M*. *chamomilla+* NaOH) sample verifies the presence of silver nanoparticles with the weight percentage of % 43.18, in addition to other peaks indicating the presence of N (% 4.18), O (% 11.20), C (% 13.98), and Cl (% 21.57).

The EDX analysis of the compound species (*C. longa*+ *M. chamomilla+* NaOH) illustrated a strong carbon signal with a weight percentage of % 41.71 and an Ag absorption peak at 3 keV with a weight percentage of % 25.98 which was not strong, and another absorption peak indicating O with the weight percentage of % 22.72 ..

The compound sample (*A. wilhelmsii + M. chamomilla +* NaOH) illustrated an Ag signal at 3 keV, verifying the presence of silver nanoparticles with a weight percentage of % 28.51 which were not strong. A strong C signal with a weight percentage of % 35.31 was detected. Moreover, other absorption peaks including N (% 4.96), K (% 4.15), and O (% 21.33) were demonstrated.

The compound sample (*A. wilhelmsii + C. longa +* NaOH) illustrated a strong signal at 3 keV verifying the presence of silver nanoparticles with a weight percentage of % 37.22; moreover, other signals of C and O were detected with the weight percentages of % 28.24 and % 22.23 respectively.

The EDX analysis of the compound sample (*A. wilhelmsii* + *C. longa* + *M. chamomilla*+ NaOH) illustrated a silver signal at 3 keV, verifying the presence of silver nanoparticles with the weight percentage of % 30.58, though not strong. A strong C signal with a weight percentage of %38.98 was detected, and another absorption peak including O with a weight percentage of % 21.67.. A silver signal at 3 keV in the compound sample (*M. chamomilla + A. wilhelmsii+ C .langa)* indicated the presence of silver nanoparticles with the weight percentage of % 32.15, which was not strong. A strong C signal with the weight percentage of % 36.05 was detected. Other absorption peaks included N (% 5.34), K (% 6.23), and O (% 15.87).

The SEM results indicated that the majority of samples had a spherical shape which verifies the presence of silver nanoparticles. Our results agreed with the results of [63] on ginger (*Z.officinael),* [9] on *C. longa,* [59] on *A*. *wilhelmsii,* [51] on *A*. *nigra,* [52] on *A*. *wilhelmsii,* [57] on *S.officinalis,* [72] on *C. longa.* In addition, the EDX results showed strong silver signals in some samples and strong carbon signals in some others. This might be due to the biomolecules absorbed into the nanoparticle layers, showing the reduction of +Ag ions to elemental Ag. The little amount of these elements in the samples could be because of the existence of biomolecules in the extract [46]. The present results agree with the results of [73] on *C .longa* and [74] on *M. chamomilla.*

### Anti-bacterial activity evaluation

#### Extracts

The analysis of the antimicrobial activity of the extracts under study against the three bacteria *Acinetobacter baumannii, Staphylococcus aureus,* and *Staphylococcus epidermidis* indicated no inhibition zone (inhibition zone diameter) against *A. baumannii* and *S. aureus*. The Analysis of variance showed that the type of extract had a significant effect on the inhibition zone against *S. epidermidis* (*P*≤ 0.01). All extracts except the compound extracts (*A. wilhelmsii+C. longa*) and (*A. wilhelmsi+M. chamomille*) showed a significant inhibition effect on *S. epidermidis* bacteria. The highest inhibition zone against this bacteria was exhibited by the compound extract (*A. wilhelmsii+C. langa+ M. chamomilla*) with an inhibition zone diameter of 10 mm. On the other hand, the results showed that the MCI and MBC values for all extracts against all the three bacteria variations were 1600 µg/mL which was significantly weak in comparison to rifampin and gentamicin (Table 3). The results showed that the most antibacterial activity was attributed to the compound extract of all three species against *S. epidermidis*. Moreover, these species did not have any inhibitory effects on the bacteria under study while the combination of these three species had an acceptable antimicrobial effect on skin bacteria.

**Table 3:** The antimicrobial properties of the extracts

Test microorganism	*A*.*wilhelmsii*			*M*. *chamomilla*			*C*. *longa*			*M.chamomilla+C.longa*			*A.wilhelmsii+ C. longa*			*M.chamomilla+ A. wilhelmsii*			*A. wilhelmsii+M.chamomilla+ C. longa*		
	DZ	MIC	MBC	DZ	MIC	MBC	DZ	MIC	MBC	DZ	MIC	MBC	DZ	MIC	MBC	DZ	MIC	MBC	DZ	MIC	MBC
*Acinetobacter baumannii*	ND	>16000	>16000	ND	>16000	>16000	ND	>16000	>16000	ND	>16000	>16000	ND	>16000	>16000	ND	>16000	>16000	ND	>16000	>16000
*Staphylococcus aureus*	ND	>16000	>16000	ND	>16000	>16000	ND	>16000	>16000	ND	>16000	>16000	ND	>16000	>16000	ND	>16000	>16000	ND	>16000	>16000
*Staphylococcus epidermidis*	9	>16000	>16000	8	>16000	>16000	8	>16000	>16000	8	>16000	>16000	ND	>16000	>16000	ND	>16000	>16000	10	>16000	>16000

DZ: inhibition zone in diameter around the well impregnated with the extracts for each microorganism. Inhibition zones values include the well diameter (6.0 mm).ND: no inhibitory effect was detected

*Staphylococcus epidermidis* can cause hospital- and community-acquired opportunist infections, among which the hospital-acquired kind is more dangerous for patients [75].

#### Synthesized nanoparticles

The ANOVA results indicated a significant difference between the inhibition zone diameter of the silver nanoparticles under study and the positive control antibiotics against the diverse variations (*P*≤0.01) (Table 4).

**Table 4:** The antimicrobial properties of nanoparticles

Test microorganism	*A.wilhelmsii+C. longa+*NaOH			*A.wilhelmsii+ C. longa+M. chamomilla+*NaOH			*C. longa+*NaOH			*M. chamomilla+*NaOH			*A.wilhelmsii+*NaOH			*A.wilhelmsiia+ M.chamomilla+ C. longa*			*M.chamomilla+ C.longa+ N*			*A. wilhelmsii+ M. chamomilla+ NaOH*		
	DZ	MIC	MBC	DZ	MIC	MBC	DZ	MIC	MBC	DZ	MIC	MBC	DZ	MIC	MBC	DZ	MIC	MBC	DZ	MIC	MBC	DZ	MIC	MBC
*Acinetobacter baumannii*	10.5	>2000	>2000	9	500	500	13	2000	2000	10	>2000	>2000	9	2000	2000	10.5	500	500	ND	500	1000	ND	500	500
*Staphylococcus aureus*	21	>2000	>2000	23	500	500	20	2000	2000	19.5	>2000	>2000	19	1000	1000	22.5	500	1000	21	1000	1000	20	500	500
*Staphylococcus epidermidis*	20	>2000	>2000	23	500	500	21	2000	2000	18	>2000	>2000	20	2000	2000	23	500	500	21.5	500	500	21.5	500	500

DZ: inhibition zone in diameter around the well impregnated with the nanoparticles for each microorganism. Inhibition zones values include the well diameter (6.0 mm).ND: no inhibitory effect was detected

The results of the Agar inhibition zone showed that the highest inhibition zone against *A. baumanni* was created by the silver nanoparticles synthesized from the (*C. longa* + NaOH) species with a 13 mm diameter. The smallest inhibition zone diameter (9 mm) was made by the silver nanoparticles synthesized from the compound species (*A. wilhelmsii+C. longa+ M. chamomilla+* NaOH) against this bacteria. The results indicated that the nanosilver MIC values against this bacteria were the least for the compound samples from the three species (*A. wilhelmsii+C. longa+ M. chamomilla*), and the two species (*A. wilhelmsii+M. chamomilla+* NaOH) at 500 µg/mL which shows a stronger performance against this bacteria compared to the other synthesized silver nanoparticles [76] found the MIC value of silver nanoparticles synthesized from *Rhaponticum repens* L. at 200 µg/mL which does not agree with our results.

Acinetobacters are one of the most successful pathogens in modern healthcare which have spread vastly in most hospitals due to the increase in surgeries, the use of antibiotics, and the presence of hosts suffering from immune deficiency. Widespread global epidemics due to antibiotic resistance and unique genetic characteristics in this organism produce factors that promote resistance in tough environments, making it a subject of research in the past two decades [77, 78].

The Gram-negative bacteria *A.baumannii* is one of the most prominent variations causing hospital-acquired infections, especially in burns and surgery intensive care, such as pneumonia complicated by ventilator bacteremia, urinary tract infection, wound, skin, and meningitis infections [79, 80].

The inhibition zone diameter study for various silver nanoparticles against *S. aureus* indicated that the highest inhibition belonged to the nanosilver synthesized from the compound extract (*A. wilhelmsii+C. longa+ M. chamomilla*) and (*A. wilhelmsii+C. longa+ M. chamomilla+*NaOH) with the inhibition zone diameter of 23 mm. The least inhibition diameter against this bacteria was recorded at 19 mm, produced by the silver nanoparticles synthesized from the (*A. wilhelmsii+* NaOH) species. Similarly, the inhibition zone diameter against this bacteria by the nanosilver synthesized from (*C. longa*) was 18 mm [72], and by the nanosilver synthesized from (*Z. officinale*. L) was 16 mm [63]. The MIC and MBC results against this bacteria showed that the nanoparticles synthesized from the compound samples (*A.wilhelmsii+M. chamomilla+* NaOH) and (*A. wilhelmsii+C. longa+ M. chamomilla+* NaOH) had the lowest number (500 µg/mL), hence the strongest performance against this bacteria [72] reported the MIC value of silver nanoparticles synthesized from *C. longa* against this bacteria to be 2500 µg/mL which disagrees with our results [81] reported that the silver nanoparticles synthesized from *Scrophularia striata* against this bacteria had an inhibition value of 31.25 µg/mL, which is far stronger than the synthesized samples in the present study [82] reported that the MIC value of AgNPs using *Arthrospira* sp polysaccharides was 3.7 μg/mL against *S. aureus*, which had acted very strongly.

The Gram-positive bacteria *S.aureus* is one of the main causes of hospital-acquired infections with increasing spread [25]. This bacteria colonizes repeatedly in the human body and is a major pathogen that can cause numerous skin infections [83]. *S.aureus* skin infections can spread from one part of the body to another and cause intense ailments [40].

The results of the inhibition zone diameter of various synthesized silver nanoparticles against *S. epidermidis* illustrated that the highest inhibition against this bacteria belonged to the nanosilver synthesized from the compound extract (*A. wilhelmsii+C. longa+ M. chamomilla+*NaOH) and (*A. wilhelmsii+C. longa+ M. chamomilla*) with the inhibition zone diameter of 23 mm. On the other hand, the smallest inhibition zone diameter against this bacteria was 18.5 mm belonging to the silver nanoparticles synthesized from the (*A*. *wilhelmsii*+ NaOH) species. The MIC and MBC of 500 µg/mL against this bacteria were illustrated by the synthesized nanoparticles from the compound samples (*A. wilhelmsii+C. longa+ M. chamomilla+*NaOH), (*A. wilhelmsii+C. longa+ M. chamomilla*), (*A. wilhelmsii*+*M.chamomilla+* NaOH), and (*C. longa*+ *M. chamomilla+* NaOH) which indicates a stronger performance from other samples.

The studies show that some bacteria show high resistance against silver nanoparticles which can be due to the thicker cell wall of Gram-positive bacteria including *S.aureus.* As Gram-negative bacteria possess a thinner wall and a layer of lipopolysaccharide with a negative charge, the reaction with silver nanoparticles, which have a weak positive charge, becomes easier. This reaction initially causes a hole in the cell wall, and when a nanoparticle enters the cell, the bacteria die. Silver nanoparticles cause the protecting parts of the cell membrane to dismantle, resulting in the release of molecules such as LPS and purines from the cytoplasmic membrane. Nanosilver not only sticks to the cell membrane but also penetrates the bacteria cell and deactivates the enzymes, producing hydrogen peroxide which kills the bacteria. Silver nanoparticles destroy cellular respiration after sticking to the cell membrane by Ag+ disrupting the enzyme-respiration chain reaction [84].

There is a relationship between the plant species, nanoparticle sizes, and the extraction method. Some of the plants have higher antimicrobial, antioxidant, and reducing properties due to more specific compounds such as flavonoids, phenols, and terpenoids. Not only does this affects the size of the synthesized nanoparticles, but the effectiveness of the silver nanoparticles also depends on their size and the extract solution type. Silver nanoparticles lower than 50 nm are stronger in penetration and thus more effective [85, 86].

The smaller the size of the nanoparticle, the higher their antibacterial effect. This has been verified by [74] on *C.longa* and [87] on *A. wilhelmsii.* Therefore, the difference in the MIC and MBC of the synthesized nanoparticles can be attributed to their size. Thus, the silver nanoparticles of the compound three species with particles smaller than 10 nm have stronger inhibitory abilities than the other synthesized silver nanoparticles. Moreover, the shape of the nanoparticles, and structural and genetic differences related to the geographical location are other reasons for the differences in the results [25].

### Evaluation of antioxidant activities of extracts

**Fig. 13:**
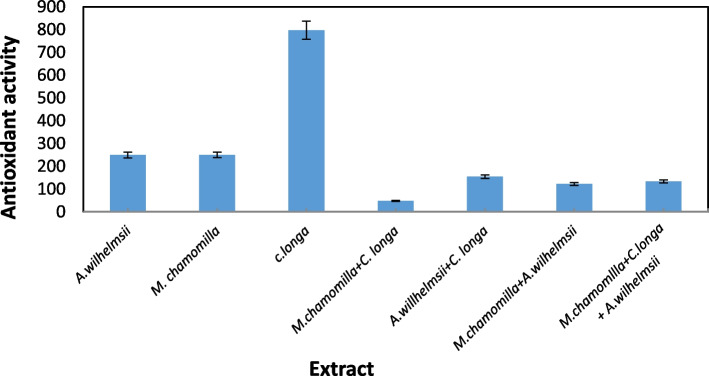
The antioxidant capacity of the extracts based on IC50

The results of the variance analysis for the antioxidant capacity based on IC50 showed that the extracttype had a significant level with an error probability of one percent (*P*≤ 0.01) (Fig. 13). The highest and lowest level of IC50 belonged to *C. longa* and *M*. *chamomilla*+ *C. longa* extracts respectively. Similarly, a relatively high antioxidant effect was reported for *C. longa* by [9], and for *A.wilhelmsii* by [59].

Prior studies indicate that the IC50 amount is inversely proportional to the antioxidant activity; thus, the lower the IC50, the stronger the antioxidant activity [88]. Consequently, *C. longa* and (*M*. *chamomilla*+ *C. longa)* extracts have the least and most antioxidant activities respectively. The studies indicate that extract secondary metabolites such as phenols and flavonoids have antioxidant activities, and prevent oxidative stress. They show their antioxidant activities mostly by binding free radicals or forming chelates with metals. Their effects increase in line with increasing the number of hydroxyl groups that are located in the phenolic rings in their structure [89]. Some plants have high abilities to biologically reduce +Ag ions to Ag0 and produce nanoparticles with antioxidant abilities [90].

One of the factors affecting the antioxidant activities of the plants is their habitat which has an impact on the formation of secondary agents via climate changes. The antioxidant compounds in the extract of a plant have multiple functions and their activity and mechanism depend on the habitat’s combination and conditions, as these can affect the synthesis of chemicals with antioxidant characteristics [91]. This antioxidant activity has been also confirmed by [92] on *Ferula assa-foetida* L. and [53] on *Fumaria officinalis*. Moreover, ecological factors such as climate, altitude, and soil characteristics are effective in antioxidant activities [48].

## Conclusion

This study was conducted to synthesize silver nanoparticles from the pure and compound extracts of the three plants *A. wilhelmsii, M. chamomilla,* and *C.longa* as an inexpensive and environmentally-friendly method, and to evaluate their antibacterial effects against some variations causing skin and wound infections, for the first time. The synthesis of almost spherical silver nanoparticles at sizes below 50 nm was verified by UV-vis, XRD, SEM, and FTTR tests. The extracts and synthesized samples had significantly diverse inhibition activities against the bacteria *S. aureus, A. baumannii,* and *S. epidermidis.* The highest inhibition zone created by silver nanoparticles synthesized from the compound extract *A*. *wilhelmsii*+ *M*. *chamomilla* + *C. longa* with size at under 10 nm against *S. epidermids* and *S. aureus* (23 mm) which was several times stronger than other extracts. Silver nanoparticles synthesized from the pure extract of *C. longa* against *A. baumannii* acted significantly stronger than other synthesized silver nanoparticles and extracts, resulting from the size and extract biomolecules. Therefore, the studied silver nanoparticles synthesized from pure and compound extracts have an environmentally-friendly nature and a potential to inhibit some skin and wound infections in nanomedicine, which indicates the need for conducting further clinical studies in the future.

## Data Availability

The datasets used and/or analysed during the current study available from the corresponding author on reasonable request.
